# Prediction of Pharmacological and Xenobiotic Responses to Drugs Based on Time Course Gene Expression Profiles

**DOI:** 10.1371/journal.pone.0008126

**Published:** 2009-12-02

**Authors:** Tao Huang, WeiRen Cui, LeLe Hu, KaiYan Feng, Yi-Xue Li, Yu-Dong Cai

**Affiliations:** 1 Institute of Systems Biology, Shanghai University, Shanghai, People's Republic of China; 2 Centre for Computational Systems Biology, Fudan University, Shanghai, People's Republic of China; 3 Key Laboratory of Systems Biology, Shanghai Institutes for Biological Sciences, Chinese Academy of Sciences, Shanghai, People's Republic of China; 4 Shanghai Center for Bioinformation Technology, Shanghai, People's Republic of China; Cairo University, Egypt

## Abstract

More and more people are concerned by the risk of unexpected side effects observed in the later steps of the development of new drugs, either in late clinical development or after marketing approval. In order to reduce the risk of the side effects, it is important to look out for the possible xenobiotic responses at an early stage. We attempt such an effort through a prediction by assuming that similarities in microarray profiles indicate shared mechanisms of action and/or toxicological responses among the chemicals being compared. A large time course microarray database derived from livers of compound-treated rats with thirty-four distinct pharmacological and toxicological responses were studied. The mRMR (Minimum-Redundancy-Maximum-Relevance) method and IFS (Incremental Feature Selection) were used to select a compact feature set (141 features) for the reduction of feature dimension and improvement of prediction performance. With these 141 features, the Leave-one-out cross-validation prediction accuracy of first order response using NNA (Nearest Neighbor Algorithm) was 63.9%. Our method can be used for pharmacological and xenobiotic responses prediction of new compounds and accelerate drug development.

## Introduction

With drug discovery now being driven primarily by bio-chemistry and high-throughput screening, the biological effects and, in particular, the pharmacology and toxicity of new compounds are required to be studied and evaluated properly before they are released. However, it is impossible to test every detail of a new compound in vitro. It is necessary to predict the possible effects of new drugs, and then experimental examinations can be initiated and orientated, resulting in a new subject of study – toxicogenomics [1], [2], [3], [4], [5] (combining the toxicology with some high-throughput technologies) – which enables us to ask some detailed questions about the possible drug effects very early on, thereby fundamentally changing the traditional approaches for the drug discovery. Microarray profiles have been used extensively in some basic biological researches, biomarker determination, pharmacology, drug target selectivity, development of prognostic tests and determination of disease-subclass, as well as in toxicogenomics. Microarray profile will also be used as the input data of pharmacological and xenobiotic response for this study. The livers play many roles in the body functioning, such as the control and synthesis of critical blood constituents including glucose, free-fatty acids, ketone bodies, amino acids, hormones, clotting factors, and inflammatory mediators [6]. The liver is critical in defense against certain infectious organisms and toxins, entered from the gastrointestinal tract [7]. Therefore, data from the liver xenobiotic and pharmacological responses are used for analysis in the study.

Both the pharmaceutical industry and the Regulatory Authorities are, despite the increasing effort to develop safer drugs, concerned by the risk of unexpected side effects observed in the later steps of the development of new drugs, either in late clinical development or after marketing approval. In order to reduce the risk of the side effects, it is important to look out for the possible xenobiotic responses at an early stage. We attempt such an effort through a prediction by assuming that similarities in microarray profiles indicate shared mechanisms of action and/or toxicological responses among the chemicals being compared [8], [9], [10] since it has been demonstrated that compounds with similar pharmacological or toxicological effects produced similar gene expression profiles either in vitro [11] or in vivo [12], [13] exposure conditions. Because one drug may have multiple responses during the regulatory time-course studies, the prediction should allow one data to be allocated to multiple classes, i.e. a multiple-target classification/prediction problem. 34 categories of pharmacological and toxicological effects were adopted (Refer to Table 1) to be the targets of each molecular compound. These categories are divided according to the body and organ weight (BO), histopathology (H), clinical pathology (CP) and structural activity class (SAC).

**Table 1:** The Characteristics of 34 responses.

Unique ID	Type	Category	Description	Drugs
SV0567082R5RU	T	CP	Absolute monocyte increase	1-NAPHTHYL ISOTHIOCYANATE, IBUPROFEN, SULINDAC, FLUCONAZOLE, NAPROXEN, ITRACONAZOLE, 4,4′-METHYLENEDIANILINE, ERYTHROMYCIN, GERANIOL, CHOLECALCIFEROL, OXICONAZOLE, CITRIC ACID, LANSOPRAZOLE, GENTIAN VIOLET, CHLOROXYLENOL, PRAZIQUANTEL, CARBAMAZEPINE, NYSTATIN, PRAMOXINE, KETOROLAC, PRALIDOXIME CHLORIDE, BENZETHONIUM CHLORIDE, ROFLUMILAST, IBUFENAC
SV0567098R5RU	T	CP	Creatinine increase	IBUPROFEN, NIMESULIDE, CISPLATIN, CHLOROFORM, LOMEFLOXACIN, FLUOXETINE, PROPYLTHIOURACIL, TICLOPIDINE, PRIMAQUINE, ANISINDIONE, SULFADIAZINE, COLISTIN, PYROGALLOL, TACRINE, ETODOLAC, ROXITHROMYCIN, AMIODARONE, NAFENOPIN
SV0567149R5RU	T	CP	Albumin increase	KETOCONAZOLE, FENOFIBRATE, LOVASTATIN, PREDNISOLONE, PRAVASTATIN, AMOXAPINE, ISONIAZID, TOLAZAMIDE, DEFERIPRONE, PRIMIDONE, MEGESTROL ACETATE, PIRINIXIC ACID, BUPROPION, BETAMETHASONE, FLUDROCORTISONE ACETATE, HYDROCORTISONE, NAFENOPIN
SV0562011R5RU	T	CP	Mean corpuscular hemoglobin concentration decrease (diagnostic, 3–7D time points)	CORTISONE, NIMETAZEPAM, THALIDOMIDE, ETODOLAC, ROXITHROMYCIN, ETHISTERONE, OXYMETHOLONE, 2,3,7,8-TETRACHLORODIBENZO-P-DIOXIN, 3-METHYLCHOLANTHRENE, PHENOBARBITAL, BETA-NAPHTHOFLAVONE, PERHEXILINE, ETHYLESTRENOL, CELECOXIB, ROFECOXIB, BENOXAPROFEN
SV0571010R5RU	T	CP	Mean corpuscular hemoglobin concentration decrease (predictive, 0.25–1D time points)	ROFECOXIB, ETODOLAC, ROXITHROMYCIN, NIMETAZEPAM, CORTISONE, THALIDOMIDE, OXYMETHOLONE, ETHISTERONE, BETA-NAPHTHOFLAVONE, 3-METHYLCHOLANTHRENE, 2,3,7,8-TETRACHLORODIBENZO-P-DIOXIN, BENOXAPROFEN, ETHYLESTRENOL, PERHEXILINE, CELECOXIB
SV0567088R5RU	T	CP	Glucose increase	KETOCONAZOLE, DEXAMETHASONE, THIOGUANINE, METHOTREXATE, CYCLOSPORIN A, CARMUSTINE, NAPROXEN, CYPROTERONE ACETATE, PROMAZINE, ISONIAZID, PYROGALLOL, BETAMETHASONE, HYDROCORTISONE, FLUOCINOLONE ACETONIDE
SV0650093R5RU	T	H	Liver- centrilobular , inflammatory cell infiltrate, mixed cell	ASPIRIN, LEFLUNOMIDE, PENICILLAMINE, CARBOPLATIN, BIS(2-ETHYLHEXYL)PHTHALATE, CHLOROFORM, CLOFIBRIC ACID, CARBIMAZOLE, AMINOSALICYLIC ACID, ISONIAZID, PYRAZINAMIDE, ACETAMINOPHEN, 3-METHYLCHOLANTHRENE, BETA-NAPHTHOFLAVONE, ALPHA-NAPHTHOFLAVONE, 2,3,7,8-TETRACHLORODIBENZO-P-DIOXIN
SV0567153R5RU	T	CP	Total protein increase	CARMUSTINE, N,N-DIMETHYLFORMAMIDE, KETOCONAZOLE, AZATHIOPRINE, CYPROTERONE ACETATE, PREDNISOLONE, PYROGALLOL, CORTISONE, ETHISTERONE, MEGESTROL ACETATE, BETAMETHASONE, FLUDROCORTISONE ACETATE, ETHYLESTRENOL, HYDROCORTISONE
SV0635003R5RU	T	CP	Leukocyte count increase	IBUPROFEN, 1-NAPHTHYL ISOTHIOCYANATE, BROMHEXINE, GENTIAN VIOLET, CHLOROXYLENOL, NYSTATIN, PRAMOXINE, TICRYNAFEN, BENZETHONIUM CHLORIDE
SV0562020R5RU	T	CP	Hemoglobin decrease	IBUPROFEN, SULINDAC, DEXAMETHASONE, THIOGUANINE, NIMESULIDE, HYDROXYUREA, CYTARABINE, INDOMETHACIN, DICLOFENAC, MELOXICAM, SULFISOXAZOLE, LIPOPOLYSACCHARIDE E. COLI O55:B5, TRICHLOROACETIC ACID, PYROGALLOL, ETODOLAC, BROMFENAC, KETOROLAC, PIOGLITAZONE, BENOXAPROFEN
SV0643003R5RU	T	BO	Relative liver weight decrease	SIMVASTATIN, ATORVASTATIN, DICLOFENAC, TAMOXIFEN, TOSUFLOXACIN, LOMEFLOXACIN, 3-METHYLCHOLANTHRENE, BETA-NAPHTHOFLAVONE, ALPHA-NAPHTHOFLAVONE, 2,3,7,8-TETRACHLORODIBENZO-P-DIOXIN, CYCLOPROPANE CARBOXYLIC ACID
SV0562050R5RU	T	CP	Alkaline phosphatase decrease	SODIUM ARSENITE, KETOCONAZOLE, METHOTREXATE, MITOMYCIN C, ETODOLAC, KETOROLAC, CYCLOPROPANE CARBOXYLIC ACID, ROFLUMILAST
SV0643002R5RU	T	BO	Relative spleen weight decrease	CHLORAMBUCIL, DEXAMETHASONE, THIOGUANINE, ROSIGLITAZONE, DOXORUBICIN, LEFLUNOMIDE, KETOCONAZOLE, METHOTREXATE, BETAMETHASONE, HYDROCORTISONE, EPIRUBICIN, FLUOCINOLONE ACETONIDE, DAUNORUBICIN, CYCLOPROPANE CARBOXYLIC ACID
SV0562014R5RU	T	CP	Mean corpuscular hemoglobin decrease (diagnostic, 3–7D time points)	ETODOLAC, ROXITHROMYCIN, 2,3,7,8-TETRACHLORODIBENZO-P-DIOXIN, 3-METHYLCHOLANTHRENE, PHENOBARBITAL, BETA-NAPHTHOFLAVONE, CYCLOPROPANE CARBOXYLIC ACID
SV0562026R5RU	T	CP	Leukocyte count decrease	CHLORAMBUCIL, VALPROIC ACID, THIOGUANINE, CYTARABINE, DOXORUBICIN, LEFLUNOMIDE, IFOSFAMIDE, CARMUSTINE, METHOTREXATE, PROCARBAZINE, MITOMYCIN C, INDOMETHACIN, ETODOLAC, EPIRUBICIN, DAUNORUBICIN, CYCLOPROPANE CARBOXYLIC ACID
SV0650033R5RU	T	H	Liver-periportal, hypertrophy	DEXAMETHASONE, ZOMEPIRAC, DICLOFENAC, MELOXICAM, MITOMYCIN C, INDOMETHACIN, MESTRANOL, ETODOLAC, KETOROLAC, CARVEDILOL, EPIRUBICIN
SV0567174R5RU	T	CP	Absolute basophil increase	3-METHYLCHOLANTHRENE, BETA-NAPHTHOFLAVONE, ALPHA-NAPHTHOFLAVONE, 2,3,7,8-TETRACHLORODIBENZO-P-DIOXIN, PERHEXILINE, ETHYLESTRENOL, CELECOXIB, ROFECOXIB, BENOXAPROFEN
SV0642001R5RU	T	BO	Relative liver weight increase	DEXAMETHASONE, ITRACONAZOLE, KETOCONAZOLE, CYPROTERONE ACETATE, ARTEMISININ, GENTIAN VIOLET, BETAMETHASONE, HYDROCORTISONE, FLUOCINOLONE ACETONIDE
SV0651106R5RU	T	H	Liver-diffuse, cytoplasm, eosinophilia	BEZAFIBRATE, FENOFIBRATE, FLUVASTATIN, CERIVASTATIN, ERYTHROMYCIN, AMINOSALICYLIC ACID, PIRINIXIC ACID, VINBLASTINE
SV0575020R5RU	T	CP	Lipase increase	ATORVASTATIN, BISPHENOL A, KETOCONAZOLE, CLOTRIMAZOLE, BITHIONOL, FLUVASTATIN, NITRAZEPAM
SV0571053R5RU	T	CP	Absolute lymphocyte decrease	CHLORAMBUCIL, THIOGUANINE, DEXAMETHASONE, DOXORUBICIN, KETOCONAZOLE, BETAMETHASONE, FLUDROCORTISONE ACETATE, HYDROCORTISONE, EPIRUBICIN, FLUOCINOLONE ACETONIDE, DAUNORUBICIN
SV0650143R5RU	T	H	Liver-periportal, fibrosis	1-NAPHTHYL ISOTHIOCYANATE, CARMUSTINE, LOMUSTINE, 4,4′-METHYLENEDIANILINE, CROTAMITON
SV0562116R5RU	T	CP	Glucose decrease	1-NAPHTHYL ISOTHIOCYANATE, CLOTRIMAZOLE, NALOXONE, BETA-NAPHTHOFLAVONE, ALPHA-NAPHTHOFLAVONE
SV0650106R5RU	T	H	Liver- hepatocyte, periportal, lipid accumulation	MICONAZOLE, ECONAZOLE, MIFEPRISTONE, ALPHA-NAPHTHOFLAVONE
SV0650121R5RU	T	H	Liver- hepatocyte, centrilobular, lipid accumulation, microvesicular	SULINDAC, MICONAZOLE, INDOMETHACIN, CHLOROFORM
SV0599196R5RU	P	SAC	GR-MR agonist	DEXAMETHASONE, PREDNISOLONE, CORTISONE, BETAMETHASONE, FLUDROCORTISONE ACETATE, HYDROCORTISONE, FLUOCINOLONE ACETONIDE
SV0614125R5RU	T	SAC	Toxicant, DNA alkylator	N-NITROSODIETHYLAMINE, HYDRAZINE, 2-ACETYLAMINOFLUORENE, 4,4′-METHYLENEDIANILINE, AFLATOXIN B1, N-NITROSODIMETHYLAMINE
SV0614137R5RU	P	SAC	Estrogen receptor agonist, steroidal	ETHINYLESTRADIOL, BETA-ESTRADIOL, BETA-ESTRADIOL 3-BENZOATE, ESTRIOL, MESTRANOL
SV0614148R5RU	P	SAC	PPAR a agonist, fibric acid	GEMFIBROZIL, BEZAFIBRATE, CLOFIBRIC ACID, PIRINIXIC ACID, NAFENOPIN
SV0599539R5RU	P	SAC	H+/K+ ATPase inhibitor	OMEPRAZOLE, PANTOPRAZOLE, LANSOPRAZOLE, RABEPRAZOLE
SV0614270R5RU	P	SAC	PDE4 inhibitor	PICLAMILAST, ROFLUMILAST, ROLIPRAM, SCH-351591
SV0599291R5RU	T	SAC	Toxicant, heavy metal (3, 5 and 7D, other non- metal toxicants in negative class)	SODIUM ARSENITE, LEAD(IV) ACETATE, LEAD (II) ACETATE
SV0614202R5RU	T	SAC	Toxicant, heavy metal (0.25–7D allowed, other toxicants not in negative class)	SODIUM ARSENITE, LEAD (II) ACETATE, LEAD(IV) ACETATE
SV0614084R5RU	P	SAC	HMG-CoA reductase inhibitors	ATORVASTATIN, FLUVASTATIN, CERIVASTATIN

The “type” column indicates the toxicity-type (T) or the pharmacology-type (P); there are four categories of responses presented, body and organ weight (BO), histopathology (H), clinical pathology (CP) and structure activity class (SAC).

Machine learning and data mining methods have been widely used in the computational biology and bioinformatics area. Many researchers have made lots of efforts to develop useful algorithms and software to investigate various biology problems such as protein post-translation modification, bio-molecular function classification, protein subcellular locations and protein-DNA interaction [14], [15], [16], [17], [18], [19], [20], [21], [22], [23], [24], [25], [26].

In this research, we present a classification of the liver toxicogenomic data [27] to support decision making of drug classification, or biomarkers when a new compound is entered for examination. The following sections will describe the microarray data obtained for the study, the analytical machine learning method which include the classification model and feature selection approach mRMR (Minimum-Redundancy-Maximum-Relevance), the results of the prediction and some discussions.

## Materials and Methods

### Original Data Set

The data used in this work are the time-series microarray data that are extracted from a large liver xenobiotic and pharmacological response database of Iconix Biosciences. The data are publicly available at GEO http://www.ncbi.nlm.nih.gov/geo under accession number GSE8858. The initial data set consists of 1695 individual animal studies and 5288 microarrays. GE Healthcare/Amersham Biosciences CodeLink UniSet Rat I Bioarray, layout EXP5280X2-584, layout EXP5280X2-613 and layout EXP5280X2-648 containing about 10000 probes was used to analyze the global gene expression in the livers of compound-treated rats. Only treatments with gene expression data of day 1, 3 and 5 were involved in our analysis, including 402 treatments with 306 compounds.

### Data Construction

First, we get a list of 10399 common probe sets between GE Healthcare/Amersham Biosciences CodeLink UniSet Rat I Bioarray, layout EXP5280X2-584, layout EXP5280X2-613 and layout EXP5280X2-648. Secondly, the gene expression profiles of 402 treatments on day 1, 3 and 5 were obtained from corresponding 3563 microarrays by averaging the duplicated experiments. Then, the control probe sets and probe sets without GenBank Accession number were excluded. The probe sets with more than 30% missing value were also excluded. This yields a subset of 9852 probes. After probe filtering, the missing expression data were imputed using nearest neighbor averaging. Finally, we normalized the expression data of 402 treatments on day 1, 3 and 5 using quantile method.

Thus expression data of 9852 genes of each day (day 1, 3 or 5) were involved in our study, producing 9852*3 = 29556 features for each of the 402 samples. Each sample is to be allocated into the 34 categories listed in Table 1, with the allowance of multiple entries into the categories, using the 29556 features.

### Minimum Redundancy Maximum Relevance Feature Selection

Minimum-Redundancy-Maximum-Relevance (mRMR) [28] is a widely used method for feature selection. The goal of mRMR is to select a feature subset that can best characterize the statistical property of a target classification variable, subject to the constraint that these features are mutually as dissimilar to each other as possible, but marginally as similar to the classification variable as possible.

The feature which has maximum relevance with the target variable and minimum redundancy within the features is defined as a “good” feature. Mutual information (MI) is defined to describe both relevance and redundancy:
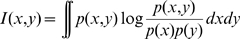
(1)Where 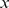 and 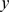 are two vectors; 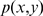 is the joint probabilistic density; 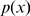 and 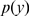 are the marginal probabilistic densities.

The whole vector set is defined as 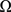, The selected vector set with 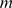 vectors is defined as 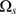, and the to-be-selected vector set with 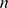 vectors is defined as 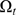. Relevance 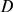 of a feature 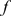 in 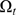 can be calculated by Eq (2):
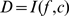
(2)Here 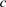 is a classification variable.

Redundancy 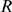 of a feature 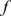 in 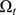 with all the features in 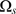 can be calculated by Eq (3):
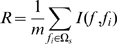
(3)mRMR function maximize relevance and minimize redundancy by integrating Eq (2) and Eq (3):
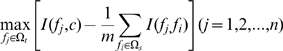
(4)After the pre-evaluation procedure, a feature set S is provided:
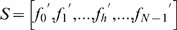
(5)the feature index reflects the evaluations for feature. The feature which fits the Eq(4) better will be added to the set S earlier. For example, If a<b, f_a_ is considered to be better than f_b_.

### Prediction Model

With the mRMR selected features, Nearest Neighbor Algorithm (NNA) [29] is used to classify the data into the above mentioned categories. NNA allocates a new data into categories by comparing the features of the data with the features of those that have known categories. The similarity between two vectors *p*_x_, *p*_y_ is defined as [25]:
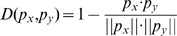
(6)where 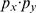 is the inner product of *p_x_* and *p_y_*, and ||*p*|| is the module of vector *p*. *p_x_* and *p_y_* are considered to be more similar if D(*p_x_*,*p_y_*) is smaller.

Traditionally, NNA chooses to classify the new pattern *p_t_* into the class of its nearest neighbor which has the smallest D(*p_n_*,*p_t_*). That is:
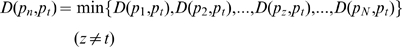
(7)where N represents the number of training samples.

Because this research is about multi-target classification i.e. a data can belong to more than one category, the prediction model needs to be adjusted to cope with the multi-target problem. In the prediction of multi-targets, if D(*p_m_*,*p_t_*)<D(*p_n_*,*p_t_*), it means that *p_t_* is closer to *p_m_* than to *p_n_*. Thus we rank the predicted classes of each drug data as:
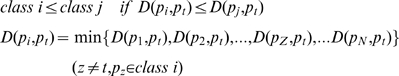
(8)From Eq. (8), we can get a list with the most likely class (defined as order-1 response) to be in the first position, and the second likely class (defined as order-2 response) to be in the second position, and so on.

### Jackknife Cross-Validation Method

Jackknife Cross-Validation Method [14], [18] is an effective and objective way to evaluate statistical predictions. Each sample in the data set is in turn knocked out and tested by the predictor trained by the other samples remaining in the data set. During the process, every sample is used not only for the training, but also for the testing. The prediction accuracy Q for overall samples was used to evaluate the performance of predictor:
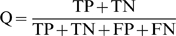
(9)where TP, TN, FP and FN stand for true positive, true negative, false positive and false negative, respectively.

### Incremental Feature Selection (IFS)

mRMR only provides a list of features by sorting the features according to their importance to the prediction without telling how many fore features in the list should be selected. The fore features are selected by testing all possible feature sets, and choosing the feature set that achieves the best prediction rate. A possible feature subset S_i_ can be expressed by the following equation.
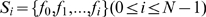
(10)The initial feature subset is 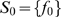, and the last feature subset is 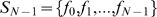 which includes all the features. Jackknife test is then used to obtain the accurate prediction rates of all the feature subsets. The one that achieves the highest prediction accuracy is considered to be optimized feature set selected by IFS. We can plot a curve, called IFS curve, with index i as its x-axis and the overall accurate rate as its y-axis.

## Results

### IFS Curves of the Drug Responses

**Figure 1:**
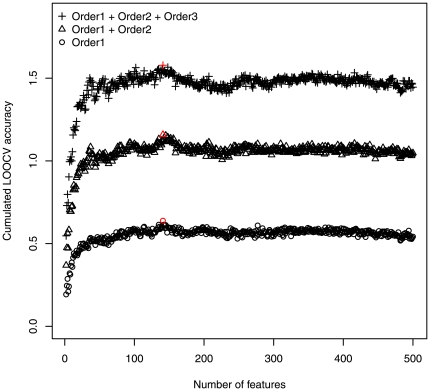
The IFS curve of first three responses prediction. The order-1 response is the most possible response according to the prediction. The highest prediction accuracy of first order response was 63.9% with 141 features. The highest cumulated prediction accuracies of first two responses and first three responses were also achieved with these 141 features. The red color points represent the highest accuracy points of each kind of accuracy.

Because a drug may have several pharmaceutical responses, Eq. 8 is used to rate all the available responses. We only take the first three responses for every drug. And more will be available if they are needed in a future research. The cumulated prediction accuracies of first one, two, and three responses using different number of features, are shown in Figure 1, evaluated by jackknife cross-validation test. The highest prediction accuracy of first order response was 63.9% with 141 features. The highest cumulated prediction accuracies of first two responses and first three responses were also achieved with these 141 features. The detailed information of the IFS procedure and these 141 features can be found in Table S1 and Table S2.

### IFS Feature Selection and the Prediction Accuracy

141 features are selected as the result according to the IFS curves. Using these 141 features, the highest prediction accuracy for the first order response is 63.9%, evaluated by jackknife cross-validation test. Unfortunately, the prediction accuracy is rather low, which might be due to the sparse data points in the high-dimensional feature space. More samples could be used in a future research to study how much the prediction accuracy is affected by the number of samples available for training and predicting the prediction model. And the biological relevance of these 141 features was explored by KEGG and GO category enrichment analysis.

The KEGG category enrichment analysis (see Table S3) shows that two of the 141 features, Cyp3a9 and Ephx1, involves in the pathway for the metabolism of xenobiotics by cytochrome P450. Cytochrome P450s (CYP), comprising a superfamily of heme-thiolate proteins, is the main metabolizing enzyme system for foreign compounds, including drugs, and has a primary role in organism protection against potential harmful assaults from the environment [30]. It is often used as biomarker to determine human exposure to environmental molecules or to predict the susceptibility to certain pathologies [31], [32].

The GO category enrichment analysis results (see Table S4, Table S5 and Table S6) show that many of these candidate biomarkers are involved in insulin signaling pathway. The insulin-mediated receptor tyrosine kinase (RTK) signaling pathways [33], [34] by downstream effectors such as phosphatidylinositol 3-kinase, mitogen activated protein kinase (MAPK), Akt/protein kinase B (PKB), mammalian target of rapamycin (mTOR), and the p70 ribosomal protein S6 kinase (p70S6 kinase) have been reviewed [35] in the regulation of drug metabolizing enzyme expression in response to insulin and growth factors. The term fatty acid metabolism, comprising genes such as fatty acid synthase, enoyl-CoA hydratase, acyl-CoA synthetase among others is also enriched. The liver is a major site for fatty acid and lipid metabolism, and several major classes of compounds appearing in the database (statins, fibrates, glitazones, estrogen receptor modulators and others) affect the lipid synthesis and degradation. Fatty acids are a major energy source and important constituents of membrane lipids, and they serve as cellular signaling molecules that play an important role in the etiology of the metabolic syndrome [36]. Some liver samples exhibited elevated triglyceride levels that were correlated with changes in the urinary associated with defective metabolism of fatty acids, confirmed by the in vitro experiments [37].

## Discussion

Microarray gene expression profiles has been proved valuable in numerous applications including disease classification, diagnosis, survival analysis, choice of therapy etc [38], but rarely used for drug response prediction. The Connectivity Map [39], [40] was a new tool for finding connections among small molecules sharing a mechanism of action, chemicals and physiological processes, and diseases and drugs. But it couldn't systematically research drug response, because the reference collection of gene-expression profiles in Connectivity Map were from cultured human cells treated with bioactive small molecules and most cells were cancer cell lines. The dataset we used were from in vivo rat liver which is closer to clinic. The compound-treated rats had same background. The bias in our research was much smaller. In the dataset of our research, the small molecules were well organized and all the responses were explicit recorded. There were thirty-four distinct pharmacological and toxicological responses. In meta-dataset like Connectivity Map's reference collection, each experiment only provided the phenotype this research group was interested in; other responses were ignored in most time.

The statistic basis of Connectivity Map wasn't solid [40]. The methods we used like mRMR and NNA have solid statistic basis and have been widely used in machine learning studies for a long time. The results were proved effective strictly using Jackknife Cross-Validation.

This paper presents a multi-target prediction for pharmacological and xenobiotic responses from drugs, i.e. allocating a drug treatment to several responses. Microarray data from liver xenobiotic and pharmacological responses are adopted for the prediction. Each drug treatment is coded by the genes of the treated subjects, derived from the microarray profile, resulting in thousands of features. Then mRMR method and IFS are used to select a compact feature set (141 features) for the reduction of feature dimension and improvement of prediction performance. Finally, the features in the compact set, considered to be most important for the prediction, are analyzed through GO category enrichment analysis.

## Supporting Information

Table S1 IFS prediction accuracy using different number of features. The first column is the number of features used in prediction. The following columns gave the prediction accuracies from order-1 (the most possible response) to order-34 (the most impossible response). The highest prediction accuracy of first order response was 63.9% with 141 features.
(0.32 MB XLS)

Table S2 The detailed information of 141 features. The first column is the feature name (probe name with time point). There are 3 time points: day 1, 3 and 5 after treatment start. The third column is the mRMR score.
(0.12 MB XLS)

Table S3 The KEGG enrichment of 141 features.
(0.01 MB XLS)

Table S4 The Gene Ontology Biological Process enrichment of 141 features.
(0.07 MB XLS)

Table S5 The Gene Ontology Molecular Function enrichment of 141 features.
(0.03 MB XLS)

Table S6 The Gene Ontology Cellular Component enrichment of 141 features.
(0.02 MB XLS)
